# PERSONALITY AND LONELINESS IN LATER LIFE: THE MEDIATING ROLE OF FRIENDSHIP

**DOI:** 10.1093/geroni/igac059.2641

**Published:** 2022-12-20

**Authors:** Emily Lim, Jeffrey Burr

**Affiliations:** University of Massachusetts Boston, Boston, Massachusetts, United States; University of Massachusetts Boston, Boston, Massachusetts, United States

## Abstract

Loneliness is prevalent among American older adults and is related to poor health outcomes. Existing studies on the association between personality and loneliness are limited and no study examined the mediating role of friendship. This study investigated how personality traits are linked to loneliness, and how friendship mediated the relationship between personality and loneliness. Using three waves of the Health and Retirement Study (N=3,259), we estimated a model with personality traits in 2010 (openness to experience, extraversion, consciousness, agreeableness, and neuroticism), friendship dimensions in 2014 (number of close friends, frequency of contact with friends, positive and negative friendship qualities), and loneliness in 2018 among community-dwelling adults aged 50 years and above (M=65.54 years old, SD=8.93). Extraversion and neuroticism were negatively and positively associated with loneliness, respectively. All friendship dimensions, except negative friendship quality, were negatively associated with loneliness. Our structural equation modeling results indicated that the number of close friends negatively mediated the relationship between extraversion and loneliness. The relationships between extraversion, agreeableness, neuroticism and loneliness were negatively mediated by positive friendship quality. Negative friendship quality positively mediated the relationship between neuroticism and loneliness, and negatively mediated the consciousness-loneliness link. Friendship contact frequency negatively mediated the relationships between openness to experience, extraversion, and loneliness but positively mediated the relationship between consciousness and loneliness. These results provided insights that help us better comprehend the mechanisms leading to loneliness. Social intervention programs could be developed that tailor different personality traits and friendship dimensions to potentially reduce loneliness in later life.

